# Network pharmacology approach to decipher signaling pathways associated with target proteins of NSAIDs against COVID-19

**DOI:** 10.1038/s41598-021-88313-5

**Published:** 2021-05-05

**Authors:** Ki Kwang Oh, Md. Adnan, Dong Ha Cho

**Affiliations:** Department of Bio-Health Convergence, College of Biomedical Science, Kangwon National University, Chuncheon, 24341 Korea

**Keywords:** Biotechnology, Computational biology and bioinformatics, Genetics, Molecular biology, Health care, Medical research

## Abstract

Non-steroidal anti-inflammatory drugs (NSAIDs) showed promising clinical efficacy toward COVID-19 (Coronavirus disease 2019) patients as potent painkillers and anti-inflammatory agents. However, the prospective anti-COVID-19 mechanisms of NSAIDs are not evidently exposed. Therefore, we intended to decipher the most influential NSAIDs candidate(s) and its novel mechanism(s) against COVID-19 by network pharmacology. FDA (U.S. Food & Drug Administration) approved NSAIDs (19 active drugs and one prodrug) were used for this study. Target proteins related to selected NSAIDs and COVID-19 related target proteins were identified by the Similarity Ensemble Approach, Swiss Target Prediction, and PubChem databases, respectively. Venn diagram identified overlapping target proteins between NSAIDs and COVID-19 related target proteins. The interactive networking between NSAIDs and overlapping target proteins was analyzed by STRING. RStudio plotted the bubble chart of the KEGG (Kyoto Encyclopedia of Genes and Genomes) pathway enrichment analysis of overlapping target proteins. Finally, the binding affinity of NSAIDs against target proteins was determined through molecular docking test (MDT). Geneset enrichment analysis exhibited 26 signaling pathways against COVID-19. Inhibition of proinflammatory stimuli of tissues and/or cells by inactivating the RAS signaling pathway was identified as the key anti-COVID-19 mechanism of NSAIDs. Besides, MAPK8, MAPK10, and BAD target proteins were explored as the associated target proteins of the RAS. Among twenty NSAIDs, 6MNA, Rofecoxib, and Indomethacin revealed promising binding affinity with the highest docking score against three identified target proteins, respectively. Overall, our proposed three NSAIDs (6MNA, Rofecoxib, and Indomethacin) might block the RAS by inactivating its associated target proteins, thus may alleviate excessive inflammation induced by SARS-CoV-2.

## Introduction

An initial outbreak of pneumonia caused by unknown etiology was first reported at Wuhan in Hubei Province, China, and alerted to the World Health Organization (WHO) by the Wuhan Municipal Health Commission on 31 December 2019^1^. Later, the infectious disease experts detected severe acute respiratory syndrome coronavirus 2 (SARS-CoV-2), can rapidly transmit from person to person through interaction or respiratory droplets^2^. As a consequence of its tremendous spread globally, WHO announced a changing level from epidemic to pandemic disease (COVID-19) on March 11, 2020^3^. Although the symptoms were identical to pneumonia, however, many COVID-19 patients showed no physical sign, thus transmitting the virus to others, as silently spread^4^.

Due to a reliable vaccine's unavailability, clinicians utilize antiviral drugs and NSAIDs as a significant viable option for COVID-19 patients^5^. A recent study has reported that the use of NSAIDs is safe for COVID-19 treatment without exposing specific adverse effects^6^. Though there is a lack of evidence whether combined NSAIDs treatment could worsen COVID-19 symptoms^7^, but researchers suggested that anti-inflammatory therapies might suppress the fatal cytokine storm of COVID-19 patients^8^. Additionally, WHO announced that no evidence of unwanted side effects was reported, particularly the risk of death with NSAIDs' administration in COVID-19 patients^9^.

NSAIDs are commonly used to treat diverse anti-inflammatory symptoms due to their excellent therapeutic efficacy^10^. For example, some evidence suggests that NSAIDs are associated with mitigating depression, bladder function recovery, reduction of psychiatric events, and a decrease in cancer risk, all of which connected directly to anti-inflammatory effects^11^. Most NSAIDs are known as inhibitors of COX-1 (Cyclooxygenase-1) or/and COX-2 (Cyclooxygenase-2) involved in the synthesis of prostaglandin and thromboxanes^12^. Recently, both COX-1 and COX-2 are expressed in inflamed tissues constitutively, and Indomethacin inhibited the two forms of COX effectively^13^. Also, Indomethacin is a potent NSAIDs against rheumatoid arthritis, used as an immune response enhancer against HIV (Human Immunodeficiency Virus), inhibiting harmful immune response induced by COX-2^13,14^. Generally, COX-1 is expressed in most normal cells, and COX-2 is induced by an abundance of physiological stimulus^15^. An animal test demonstrated that the inhibition of COX-1 up-regulates COX-2 expression level, results in the prevention of aggravated effects against inflammatory response^16^. It was reported that the administration of dual COX-1/2 inhibitors slightly diminished viral DNA replication but could not induce viral DNA cleavage^17^. Hence, COX-1/2 inhibitors are involved in host immune responses while cannot take effect on pathogens. However, one potential drug is Indomethacin, which possesses both anti-inflammatory and antiviral properties. Its antiviral potentiality was first identified in 2006 during the outbreak of SARS-CoV^18^, and subsequent attribution was also observed against SARS-CoV-2^19^. A study on canine coronavirus (in vitro) revealed that Indomethacin could significantly suppress virus replication, thus protecting host cell from virus-induced damage. A similar antiviral effect was also observed during in vivo assessment where an average anti-inflammatory dose was found very effective^19,20^. Although many NSAIDs may have possible therapeutic interventions against COVID-19, but lack of scientific evidence has limited their broad application to COVID-19 patients. Hence, we aimed to identify the most influential NSAIDs and their mechanism(s) against COVID-19 through network pharmacology.

**Figure 1:**
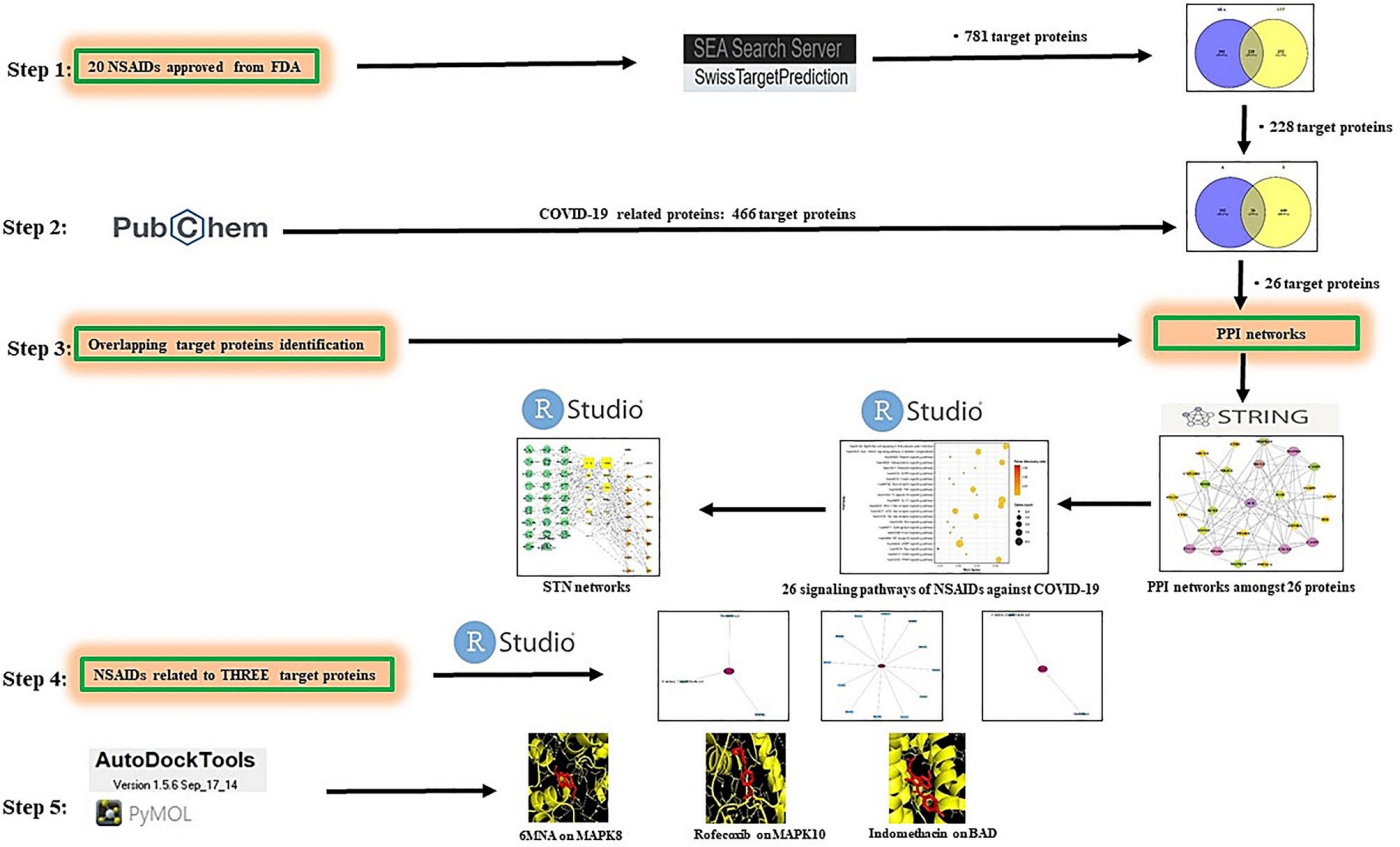
Workflow of network pharmacology analysis of NSAIDs against COVID-19.

Network pharmacology can decode the mechanism(s) of drug action with an overall viewpoint^21^, which focuses on pattern changing from "single protein target, single drug" to "multiple protein targets, multiple drugs"^22,23^. Currently, network pharmacology has been extensively utilized to explore multiple targets and unknown additional mechanism(s) against diverse diseases^24^. In this research, network pharmacology was applied to investigate the most potent NSAIDs and their novel mechanisms of action against COVID-19. Firstly, FDA approved NSAIDs (nineteen active drugs and one prodrug) were selected via using public websites. The selected NSAIDs and COVID-19 related target proteins were also identified using public databases. Next, the extracted overlapping target proteins were discovered as target proteins for analyzing anti-COVID-19 properties. Finally, pathway enrichment analysis was performed to reveal the mechanism(s) of the most potent NSAIDs against COVID-19. Figure 1 shows the overall workflow.

## Results

### Information of NSAIDs

**Figure 2:**
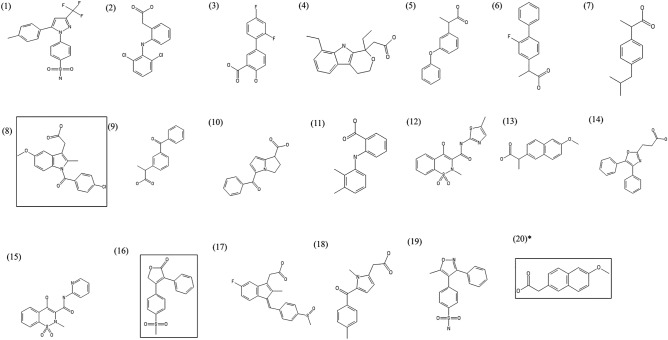
Structure of 19 NSAIDs and 1 prodrug. (1) Celecoxib (2) Diclofenac (3) Diflunisal (4) Etodolac (5) Fenoprofen (6) Flubiprofen (7) Ibuprofen (8) Indomethacin (9) Ketoprofen (10) Ketorolac (11) Mefenamic acid (12) Meloxicam (13) Naproxen (14) Oxaprozin (15) Piroxicam (16) Rofecoxib (17) Sulindac (18) Tolmetin (19) Valdecoxib *(20): Nabumetone (prodrug of 6MNA). The three NSAIDs in box line are the most potent NSAIDs candidates against COVID-19.

A total of nineteen NSAIDs and one prodrug (FDA approved) were selected. Table 1 displayed the NSAIDs' chemical information and TPSA (Topological Polar Surface Area). Among the selected NSAIDs, nineteen NSAIDs were found as an active drug, and one "nabumetone" was a prodrug, and its metabolite form is 6-methoxy-2-naphthylacetic acid (6MNA). Figure 2 exhibited the chemical structure of these NSAIDs.

**Table 1:** A list of NSAIDs (19 active drugs and one prodrug) approved by FDA and TPSA (Å^2^) values.

No	Drug name	PubChem CID	Mechanism of action	TPSA (< 140 Å^2^)
1	Flubiprofen	3394	Nonselective COX inhibitor	37.30
2	Ibuprofen	3672	Nonselective COX inhibitor	37.30
3	Indomethacin	3715	Nonselective COX inhibitor	68.53
4	Ketorolac	3826	Nonselective COX inhibitor	59.30
5	Mefenamic acid	4044	Nonselective COX inhibitor	49.33
6	Piroxicam	54,676,228	Nonselective COX inhibitor	107.98
7	Diflunisal	3059	Prostaglandin synthesis inhibitor	57.53
8	Fenoprofen	3342	Prostaglandin synthesis inhibitor	46.53
9	Naproxen	156,391	Prostaglandin synthesis inhibitor	46.53
10	Sulindac	1,548,887	Prostaglandin synthesis inhibitor	73.58
11	Tolmetin	5509	Prostaglandin synthesis inhibitor	59.30
12	Ketoprofen	3825	Selective COX-1 inhibitor	54.37
13	Oxaprozin	4614	Selective COX-1 inhibitor	63.33
14	Celecoxib	2662	Selective COX-2 inhibitor	86.36
15	Rofecoxib	5090	Selective COX-2 inhibitor	68.82
16	Valdecoxib	119,607	Selective COX-2 inhibitor	94.57
17	Diclofenac	3033	Selective COX-2 inhibitor	49.33
18	Etodolac	3308	Selective COX-2 inhibitor	62.32
19	Meloxicam	54,677,470	Selective COX-2 inhibitor	136.22
20^a^	**6MNA**	32,176	Selective COX-2 inhibitor	46.53

^a^6MNA (Active form) of Nabumetone (Prodrug); TPSA (Topological Polar Surface Area).

### NSAIDs connected to the 781 target proteins or COVID-19 targeted proteins

**Figure 3:**
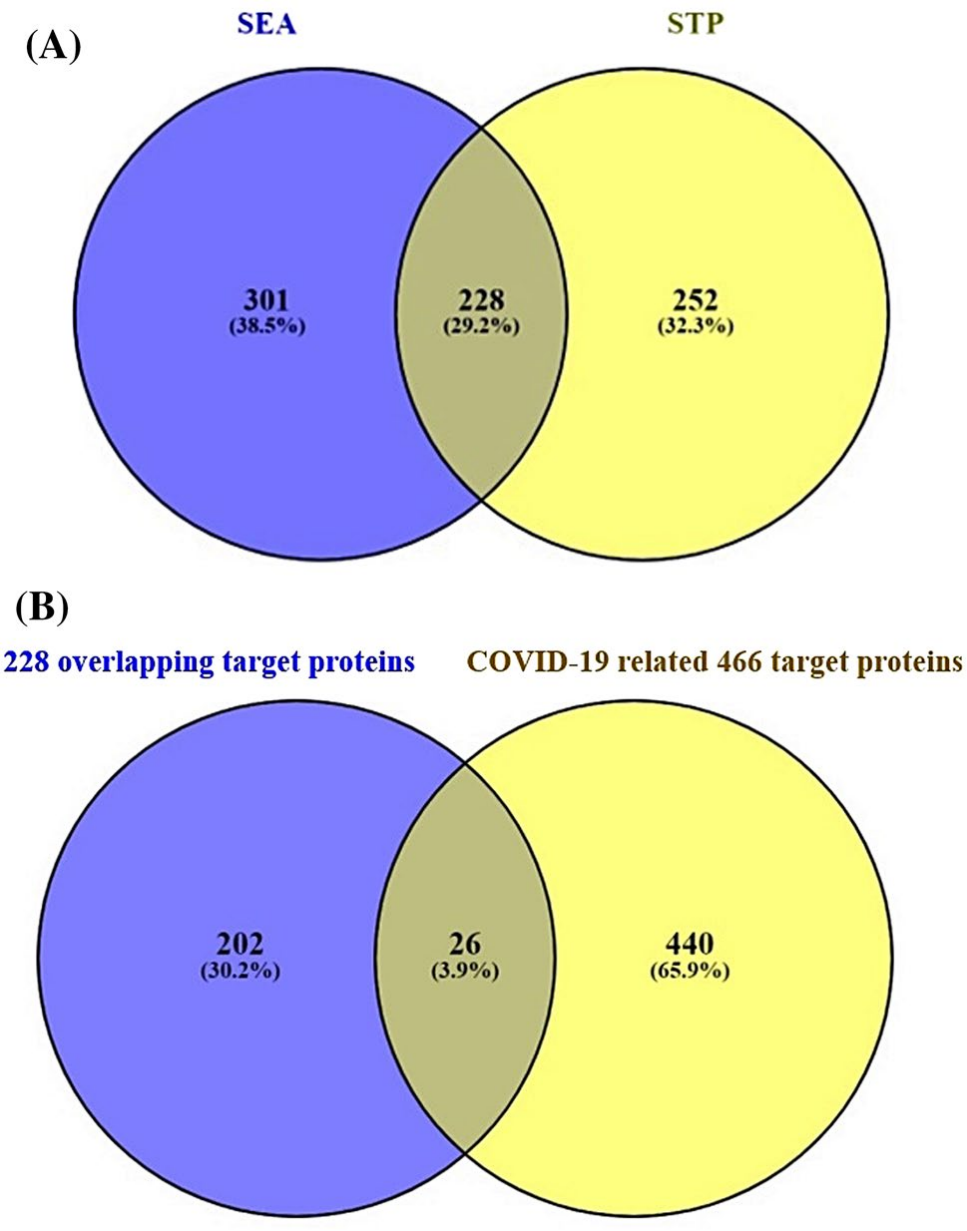
(**A**) Overlapping target proteins (228 target proteins) of NSAIDs related target proteins identified from SEA (529 target proteins) and STP (480 target proteins). (**B**) Overlapping target proteins (26 target proteins) between NSAIDs related 228 overlapped target proteins and COVID-19 related 466 target proteins.

A total of 781 NSAIDs related proteins were identified (Supplementary Table S1) through two public databases (SEA and STP). Figure 3A showed the overlapping target proteins (228 target proteins) selected from the two databases (Supplementary Table S2). The number of 466 COVID-19 targeted proteins was identified from the PubChem database (Supplementary Table S3). Figure 3B illustrated that the final 26 overlapping target proteins were selected between 228 overlapped target proteins and 466 COVID-19 targeted proteins (Supplementary Table S4).

### Protein–protein interaction (PPI) networks and KEGG pathway enrichment analysis

**Figure 4:**
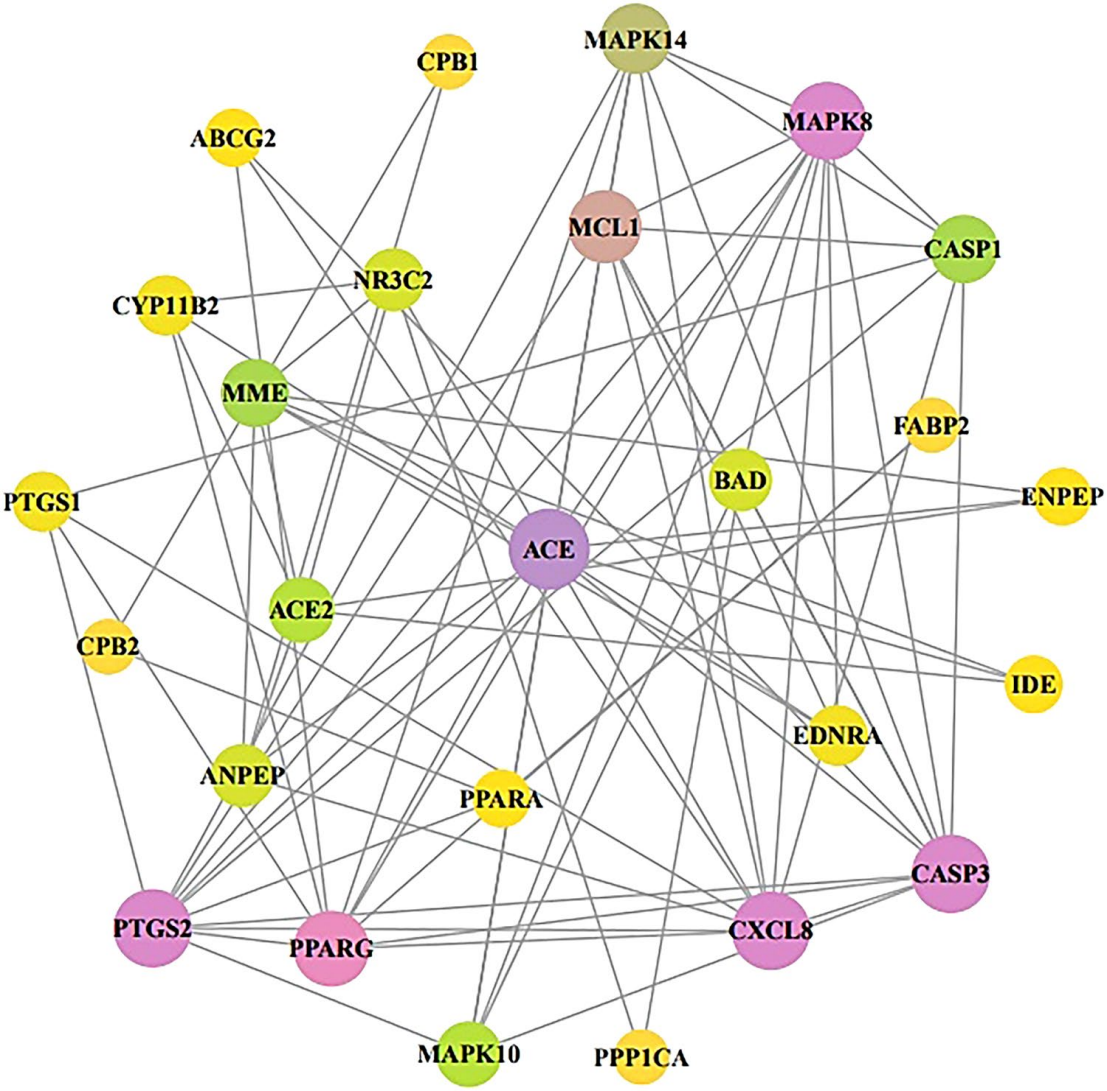
Protein protein interaction (PPI) networks with 26 nodes and 78 edges in NSAIDs against COVID-19 via STRING analysis. Node: the number of units; Edge: the number of interactions between the units.

**Figure 5:**
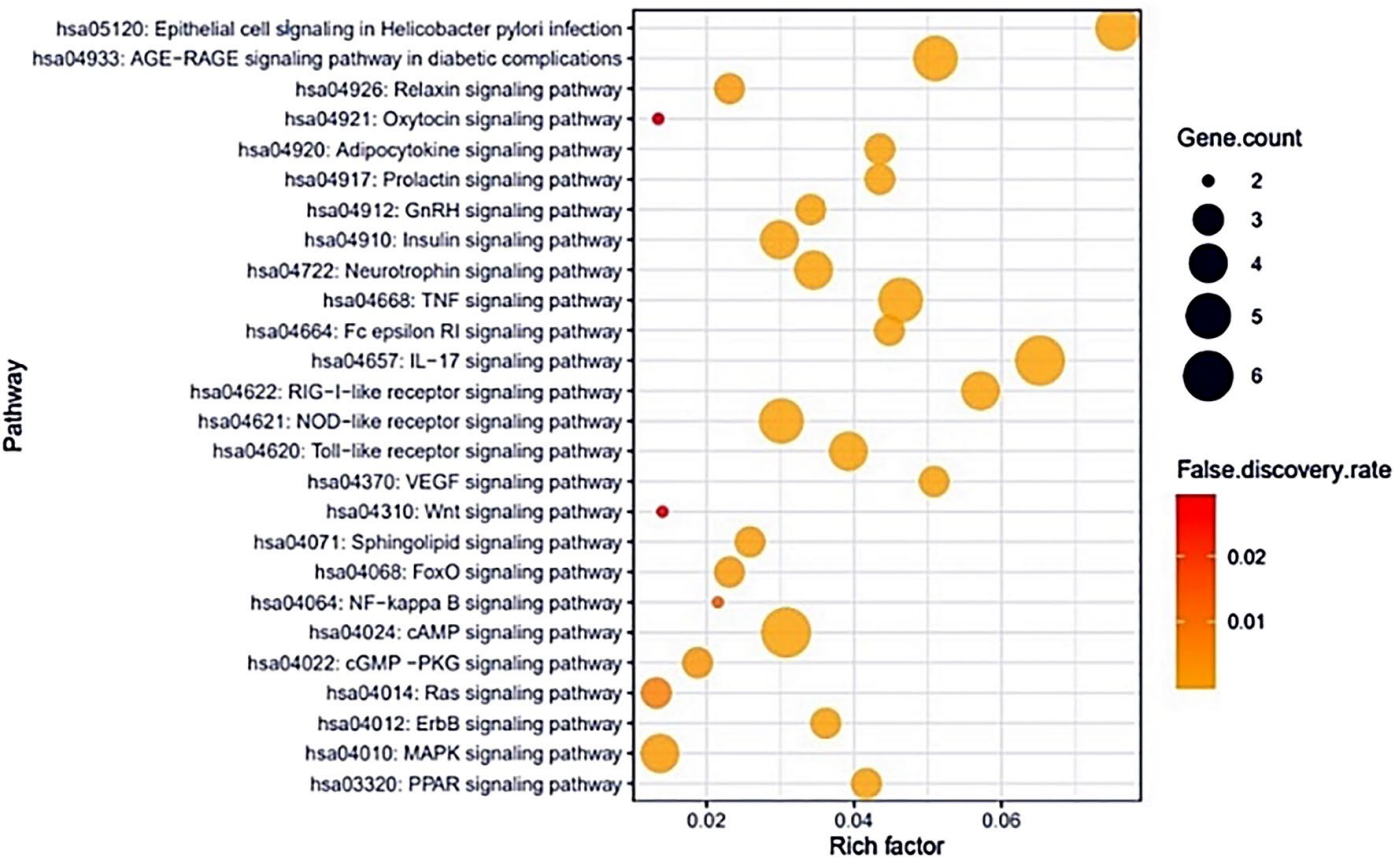
Bubble chart of 26 signaling pathways related to the occurrence and progression of COVID-19.

Figure 4 demonstrated that the final 26 overlapping target proteins were selected by STRING, which represents 26 nodes and 78 edges. According to the KEGG pathway enrichment analysis, 13 target proteins were connected to 26 signaling pathways (False Discovery Rate < 0.05). Table 2 showed the description of 26 signaling pathways. Figure 5 displayed that 13 out of the final 26 overlapping target proteins were strongly associated with 26 signaling pathways against COVID-19; moreover, the RAS signaling pathway with the lowest Rich factor was identified as a hub signaling pathway.

**Table 2:** Target proteins in 26 signaling pathways enrichment related to COVID-19.

KEGG ID & description	Target proteins	Rich factor	False discovery rate
hsa04014: Ras signaling pathway	MAPK8, MAPK10, BAD	0.013157895	0.0071
hsa04921: Oxytocin signaling pathway	PTGS2, PPP1CA	0.013422819	0.0294
hsa04010: MAPK signaling pathway	MAPK8, MAPK10, MAPK14, CASP3	0.013651877	0.0016
hsa04310: WNT signaling pathway	MAPK8, MAPK10	0.013986014	0.0276
hsa04022: cGMP -PKG signaling pathway	ENDRA, BAD, PPP1CA	0.01875	0.003
hsa04064: NF-kappa B signaling pathway	CXCL8, PTGS2	0.021505376	0.0136
hsa04926: Relaxin signaling pathway	MAPK8, MAPK10, MAPK14	0.023076923	0.0018
hsa04068: FoxO signaling pathway	MAPK8, MAPK10, MAPK14	0.023076923	0.0018
hsa04071: Sphingolipid signaling pathway	MAPK8, MAPK10, MAPK14	0.025862069	0.0014
hsa04910: Insulin signaling pathway	MAPK8, MAPK10, BAD, PPP1CA	0.029850746	0.00013
hsa04621: NOD-like receptor signaling pathway	MAPK8, MAPK10, MAPK14, CXCL8, CASP1	0.030120482	0.0000197
hsa04024: cAMP signaling pathway	ENDRA, MAPK8, BAD, MAPK10, PPP1CA, PPARA	0.030769231	0.00000373
hsa04912: GnRH signaling pathway	MAPK8, MAPK10, MAPK14	0.034090909	0.00072
hsa04722: Neurotrophin signaling pathway	MAPK8, MAPK10, MAPK14, BAD	0.034482759	0.00000853
hsa04012: ErbB signaling pathway	MAPK8, MAPK10, BAD	0.036144578	0.00062
hsa04620: Toll-like receptor signaling pathway	MAPK8, MAPK10, MAPK14, CXCL8	0.039215686	0.0000581
hsa03320: PPAR signaling pathway	PPARA, PPARG, FABP2	0.041666667	0.00044
hsa04920: Adipocytokine signaling pathway	MAPK8, MAPK10, PPARA	0.043478261	0.00041
hsa04917: Prolactin signaling pathway	MAPK8, MAPK10, MAPK14	0.043478261	0.00041
hsa04664: Fc epsilon RI signaling pathway	MAPK8, MAPK10, MAPK14	0.044776119	0.00039
hsa04668: TNF signaling pathway	MAPK8, MAPK10, MAPK14, CASP3, PTGS2	0.046296296	0.00000413
hsa04370: VEGF signaling pathway	MAPK8, MAPK10, BAD	0.050847458	0.00029
hsa04933: AGE-RAGE signaling pathway in diabetic complications	MAPK8, MAPK10, MAPK14, CXCL8, CASP1	0.051020408	0.00000373
hsa04622: RIG-I-like receptor signaling pathway	MAPK8, MAPK10, MAPK14, CXCL8	0.057142857	0.0000197
hsa04657: IL-17 signaling pathway	MAPK8, MAPK10, MAPK14, CXCL8, CASP3, PTGS2	0.065217391	0.000000135
hsa05120: Epithelial cell signaling in Helicobacter pylori infection	MAPK8, MAPK10, MAPK14, CXCL8, CASP3	0.075757576	0.000000954

### A signaling pathway-target protein—NSAID (STN) network analysis

**Figure 6:**
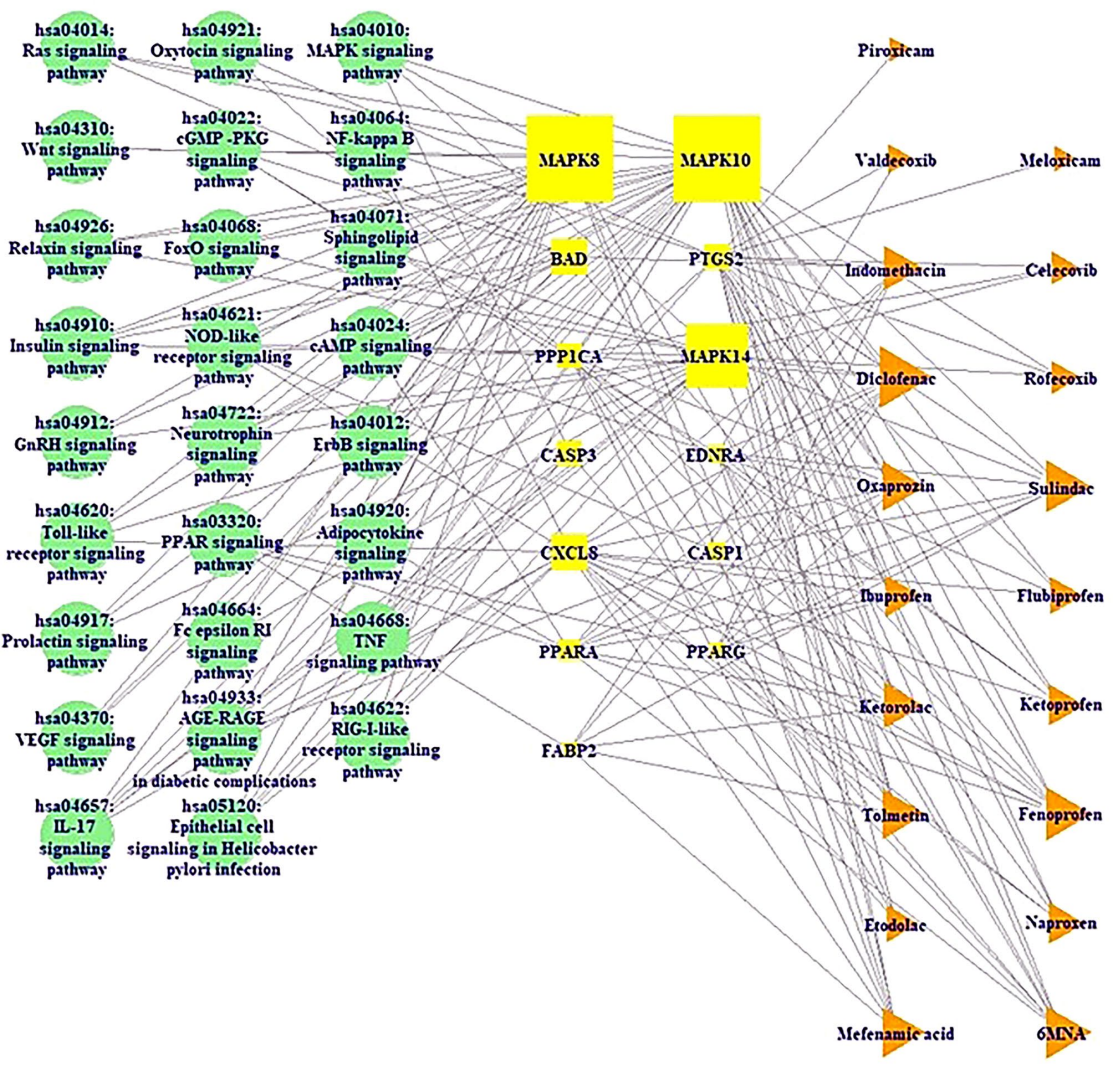
Signaling pathway-Target protein-NSAID (STN) networks. Green circle: signaling pathway; Yellow square: target protein; Orange triangle: NSAID.

Figure 6 revealed 26 signaling pathways—13 target proteins—19 NSAIDs networks (58 nodes and 194 edges). Among 20 NSAIDs, Diflunisal has no association with signaling pathways against COVID-19.

The nodes indicated a total number of signaling pathways, target proteins, and NSAIDs. The edges represented the relationships of the three components. The STN relationship suggested that the network might be potential therapeutic efficacy against COVID-19. The STN network displayed that 13 target proteins associated with 26 signaling pathways built by a size map. Among the 13 target proteins, both MAPK8 and MAPK10 have the highest degree (22), followed by MAPK14 (15), BAD (7), and CXCL8 (7). Additionally, among the 19 NSAIDs, Diclofenac has the greatest degree (10), followed by 6MNA (9), Fenoprofen (8), and Sulindac (8).

### MDT of 3 target proteins and 15 NSAIDs associated with RAS signaling pathway

**Figure 7:**
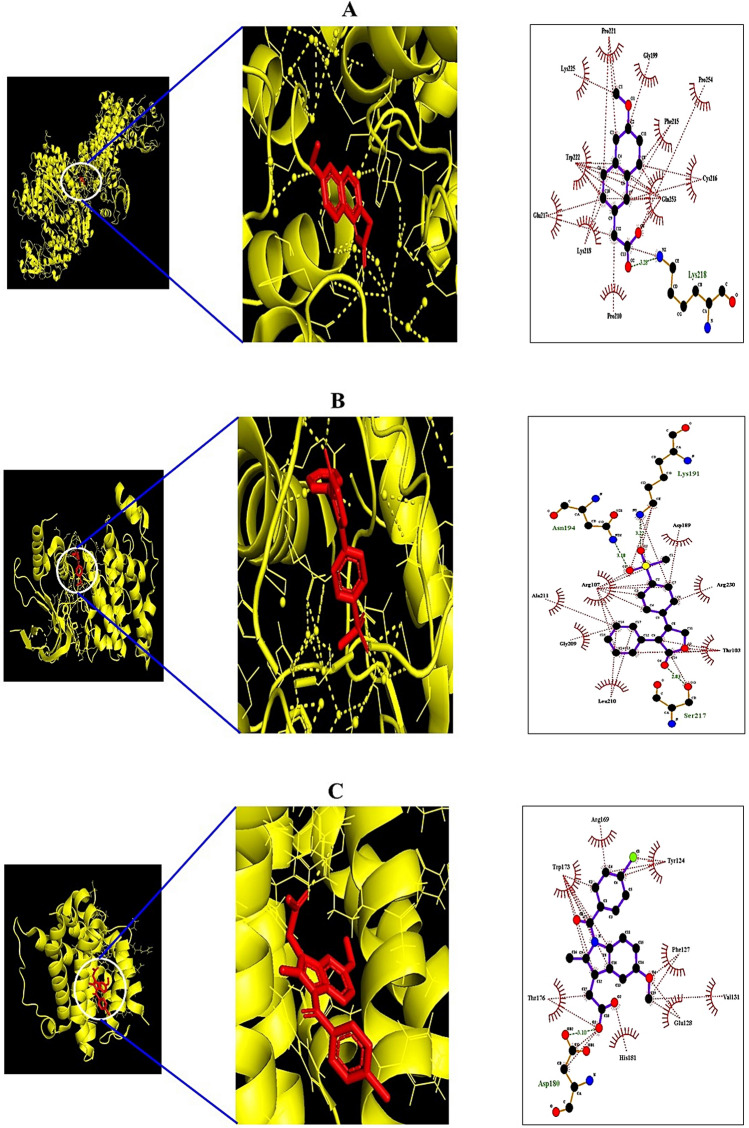
Molecular docking interaction between best docked NSAIDs and target proteins. (**A**) 6MNA on MAPK 8 (PDB ID:4YR8) (**B**) Rofecoxib on MAPK 10 (PDB ID:3TTJ). (**C**) Indomethacin on BAD (PDB ID: 1G5J).

**Figure 8:**
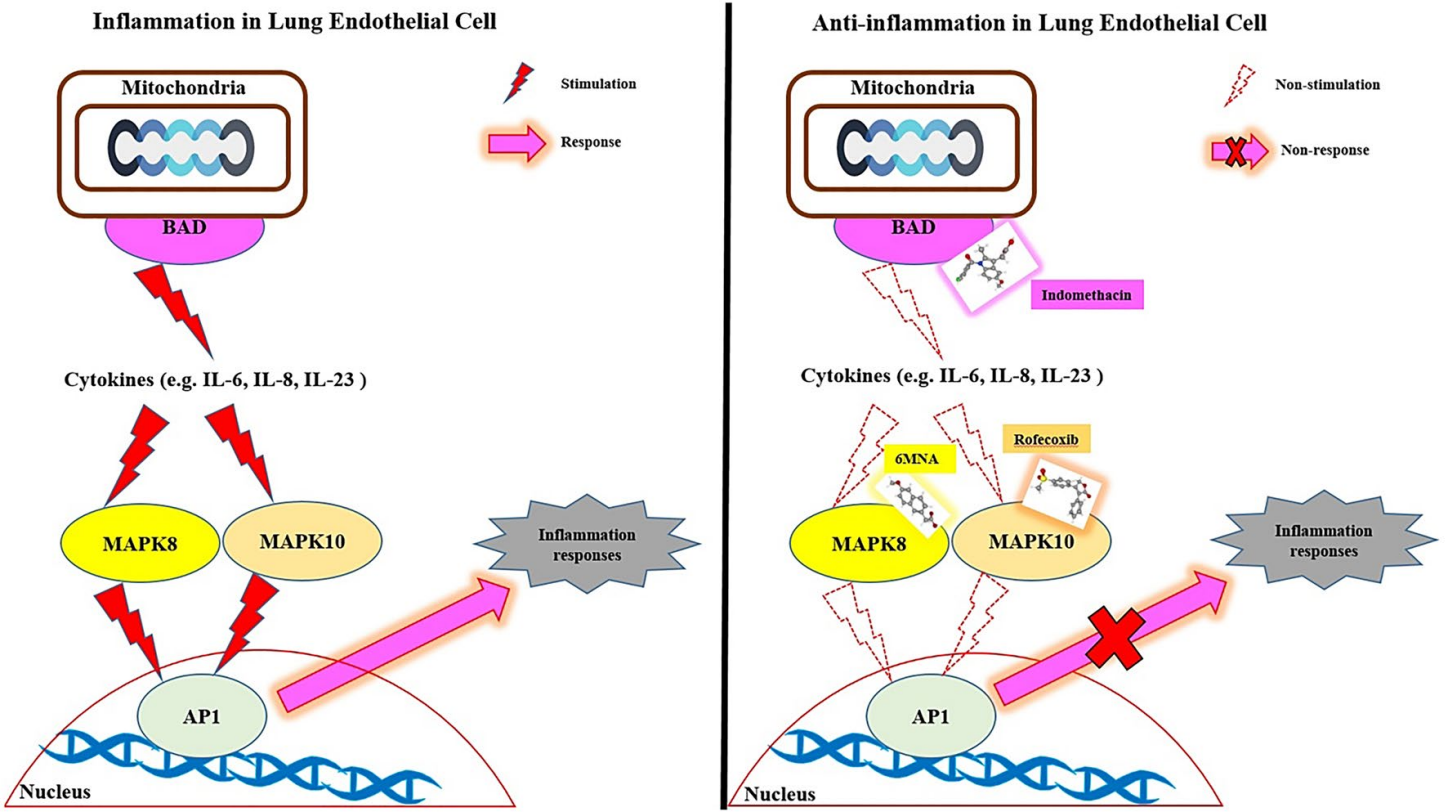
Anti-inflammation mechanisms of promising NSAIDs against COVID-19.

From the SEA and STP databases, it was revealed that MAPK8 was associated with three NSAIDs (6MNA, Mefenamic acid, and Etodolac), MAPK10 was related to twelve NSAIDs (Mefenamic acid, Naproxen, Tolmetin, Fenoprofen, Ketorolac, Ketoprofen, Ibuprofen, Flurbiprofen, Oxaprozin, Sulindac, Diclofenac, and Rofecoxib), BAD was involved with two NSAIDs (6MNA and Indomethacin). The MDT was performed to evaluate NSAIDs' binding affinity on target three proteins (MAPK8, MAPK10, and BAD) connected directly to the RAS signaling pathway. The MDT score of three NSAIDs on MAPK8 (PDB ID: 4YR8) was analyzed in the "*Homo Sapiens*" mode. Table 3 indicated that 6MNA (− 7.1 kcal/mol) revealed the highest binding energy, followed by Mefenamic acid (− 6.4 kcal/mol), and Etodolac (− 6.3 kcal/mol) on MAPK8 (PDB ID: 4YR8). Figure 7A exhibited the MDT of the 6MNA-MAPK8 (PDB ID: 4YR8) complex with the highest binding affinity. The MDT score of twelve NSAIDs on MAPK10 (PDB ID: 3TTJ) was analyzed in the "*Homo Sapiens*" mode. Table 4 indicated that Rofecoxib (− 7.5 kcal/mol) exposed the highest binding energy, followed by Sulindac (− 7.4 kcal/mol), Oxaprozin (− 7.1 kcal/mol), Ketorolac (− 7.1 kcal/mol), Flubiprofen (− 6.9 kcal/mol), Tolmetin (− 6.7 kcal/mol), Diclofenac (− 6.7 kcal/mol), Fenoprofen (− 6.5 kcal/mol), Ketoprofen (− 6.5 kcal/mol), Mefenamic acid (− 6.4 kcal/mol), Naproxen (− 6.1 kcal/mol), Ibuprofen (− 5.6 kcal/mol) on MAPK10 (PDB ID: 3TTJ). Figure 7B presented the MDT of the Rofecoxib—MAPK10 (PDB ID: 3TTJ) complex with the highest binding affinity. The MDT score of two NSAIDs on BAD (PDB ID: 1G5J) was analyzed in the "*Homo Sapiens*" mode. Figure 7C exhibited the MDT of the Indomethacin—BAD (PDB ID: 1G5J) complex with the highest binding affinity. Table 5 showed that Indomethacin (− 7.1 kcal/mol) was the highest binding energy, followed by 6MNA (− 6.8 kcal/mol) on BAD (PDB ID: 1G5J). Figure 8 depicted that MAPK-6MNA complex and MAPK10-Rofecoxib complex inhibit AP1 induced inflammatory responses; simultaneously, blocked AP1 inhibits cytokine productions. As an upstream region, the BAD-Indomethacin complex also interrupts cytokine production against COVID-19.

**Table 3:** Binding energy and interactions of potential three NSAIDs on MAPK8 (PDB ID:4YR8).

Protein	Ligand	PubChem ID	Symbol	Binding energy (kcal/mol)	Hydrogen bond interactions	Hydrophobic interactions
					Amino acid residue	Amino acid residue
4YR8	6MNA	32,176	M1	− 7.1	Lys-218	Pro-221, Gly-199
						Pro-254, Phe215
						Cys-216, Gln-253
						Pro-210, Lys-218
						Glu-217, Lys-225
	Mefenamic acid	4044	M2	− 6.4	Glu-217	Trp-222, Val-211
						Arg-208, Cys-216
						Asn-193, Lys-218
						Pro-211
	Etodolac	3308	M3	− 6.3	n/a	Tyr-202, Lys-203
						Met-200, Gly-201
						Pro-221, Lys-218
						Lys-251, Ser-307
						Ala-306

**Table 4:** Binding energy and interactions of potential twelve NSAIDs on MAPK10 (PDB ID: 3TTJ).

Protein	Ligand	PubChem ID	Symbol	Binding energy (kcal/mol)	Hydrogen bond interactions	Hydrophobic Interactions
					Amino acid residue	Amino acid residue
3TTJ	Mefenamic acid	32,176	R1	− 6.4	Arg-107	Asp-207, Gln-75
						Leu-206, Lys-93
						Asn-194, Asp-207
	Naproxen	4044	R2	− 6.1	Asn-194, Lys-93	Arg-107, Asp-189
						Val-225, Lys-191
						Gln-75, Gly-73
	Tolmetin	3308	R3	− 6.7	Asn-194, Asp-189	Lys-106, Leu-210
						Ala-211, Arg-110
						Arg-230, Lys-191
						Arg-107, Thr-103
	Fenoprofen	3342	R4	− 6.5	Lys-93, Lys-191	Ser-193, Ser-72
					Asn-194	Val-78, Gly-73
						Gln-75, Ala-74
						Arg-107
	Ketorolac	3826	R5	− 7.1	Glu-111, Arg-107	Asp-207, Leu-206
					Asn-194, Lys-93	Gln-75, Ser-193
						Ser-72, Val-78
						Gly-73
	Ketoprofen	3825	R6	− 6.5	Lys-93, Asn-194	Val-78, Leu-206
					Ser-193	Arg-107, Gln-75
						Gly-73
	Ibuprofen	3672	R7	− 5.6	Lys-93, Ser-193	Leu-206, Ala-74
					Asn-194	Gly-73, Gln-75
						Val-78
	Flubiprofen	3394	R8	− 6.9	Lys-191, Asp-189	Lys-93, Val-78
					Asn-194	Gly-73, Arg-107
						Gln-75
	Oxaprozin	4614	R9	− 7.1	Asn-194, Arg-107	Asp-189, Thr-103
					Lys-191	Ser-217, Val-225
						Arg-230
	Sulindac	1,548,887	R10	− 7.4	Asn-152	Arg-107, Asn-194
						Lys-93, Ser-72
						Gly-73, Ser-193
						Ala-74
	Diclofenac	3033	R11	− 6.7	Asn-194	Ser-72, Gly-73
						Ser-193, Gln-75
						Arg-107, Lys-93
						Leu-206, Val-78
						Gly-71
	Rofecoxib	5090	R12	− 7.5	Asn-194, Lys-191	Asp-189, Arg-230
					Ser-217	Thr-203, Leu-210
						Gly-209, Ala-211
						Arg-107

**Table 5:** Binding energy and interactions of potential two NSAIDs on BAD (PDB ID: 1G5J).

Protein	Ligand	PubChem ID	Symbol	Binding energy (kcal/mol)	Hydrogen bond interactions	Hydrophobic interactions
					Amino acid residue	Amino acid residue
1G5J	6MNA	32,176	B1	− 6.8	Trp-173, His-181	Arg-169, Tyr-124
						Phe-127, Tyr-177
						Thr-176
	Indomethacin	3715	B2	− 7.1	Asp-180	Arg-169, Tyr-124
						Phe-127, Val-131
						Glu-128, His-181
						Thr-176, Trp-173

## Discussion

STN networking analysis demonstrated that the therapeutic effect of NSAIDs against COVID-19 was directly related to 26 signaling pathways—13 target proteins—19 NSAIDs. The results of the KEGG pathway enrichment analysis of 13 target proteins suggested that 26 signaling pathways were associated with the occurrence and development of the COVID-19 symptoms. The relationships of 26 signaling pathways with COVID-19 symptoms were succinctly discussed as follows. PPAR (Peroxisome Proliferator-Activated Receptor) signaling pathway: a report shows that PPARγ (Peroxisome Proliferator-Activated Receptor-gamma), PPARα (Peroxisome Proliferator-Activated Receptor-alpha), and PPARβ/δ (Peroxisome Proliferator-Activated Receptor-beta/delta) agonists have anti-inflammatory and immunomodulatory functions^25^. MAPK (Mitogen-Activated Protein Kinase) signaling pathway: The mechanisms of p38 MAPK inactivation might be an effective therapy against the SARS infected cells^26^. Additionally, MAPK stimulates cytokine production such as IL-10 (Interleukin 10), TNF-α (Tumor Necrosis Factor-Alpha), IL-4 (Interleukin 4), and IFN-γ (Interferon-gamma)^27^. It is evident that MAPK inhibitor can alleviate inflammatory responses against COVID-19. ErbB (Erythroblastic Leukemia Viral Oncogene Homolog) signaling pathway: ErbB signaling reduces the proinflammatory activation in cardiac cells^28^. RAS (Renin-Angiotensin System) signaling pathway: The inhibition of ACE (Angiotensin Converting Enzyme) connected to RAS signaling pathway could reduce tissue damage in COVID-19 patients^29^. Thus, the study indicates that blocking of the RAS signaling pathway can reduce inflammatory response level. cGMP-PKG (Cyclic GMP-Protein Kinase G) signaling pathway: the activation of cGMP-PKG signaling inhibits inflammatory response in the prostate, and also decreases CCL5 (C–C Motif Chemokine Ligand 5) released in CD8 ^+^ T cells (Cluster of Differentiation 8 T cells)^30^. cAMP (Cyclic Adenosine Monophosphate) signaling pathway: the elevation of cAMP leads to diverse cellular effects, such as airway smooth muscle relaxation, repressed effects on cellular inflammation, and immune responses^31^. NF-κB (Nuclear Factor kappa-light-chain-enhancer of activated B cells) signaling pathway: activation of the NF-κB signaling pathway gives rise to the inflammation induced by the SARS-CoV infection. In contrast, NF-κB inhibitors are the potential antivirals, even against SARS-CoV and can also contribute to other pathogenic human coronaviruses^32^. FOXO (Forkhead box protein O1) signaling pathway: decrease of FOXO3 (Forkhead box protein O3) in T cells inhibits apoptosis, enhances multifunction of CD8 cells, and elevates viral control^33^. Sphingolipid signaling pathway: Sphingolipids play a vital role to protect the lung from damages, and its control may give a good therapeutic efficacy^34^. WNT (Wingless/Integrated) signaling pathway: WNT signaling involves with the prime inflammatory pathways like intestinal inflammation. Also, the understanding mechanism of WNT ligands and cytokines manifest new treatment strategies for chronic colitis and other inflammatory diseases^35^. VEGF (Vascular Endothelial Growth Factor) signaling pathway: a report suggested that the activation of ACE2 (Angiotensin-Converting Enzyme 2) inhibits VEGFA (Vascular Endothelial Growth Factor A), which elevates vascular permeability and severity of endothelial damage^36^. TLR (Toll-like receptor) signaling pathway: Toll-like receptors (TLRs) play a pivotal role in the innate immune system and contribute to defending host cells by recognizing PAMPs (Pathogen-Associated Molecular Patterns) induced by various microbes^37^. TLRs' activation triggers an array of responses resulting in the expression of different cytokines and chemokines, phagocytosis, and even apoptotic case activation to induce programmed cell death^38^. NOD-like receptor (NLR) signaling pathway: Nod-like receptors (NLRs) have been revealed as the major microbial signals that take part in the universal immune responses to infection and contribute to the prevention of infections^39^. RIG-I-like receptor (RLR) signaling pathway: RIG-I-like receptors (RLRs) play a vital role in the pathogen sensor of RNA virus infection, enhancing the antiviral immunity by sensing foreign RNA^40^. IL-17 (Interleukin-17) signaling pathway: IL-17 receptor inhibitors are widely used to ameliorate the inflammatory acuteness to date. Furthermore, it is a potential target to suppress severe inflammation induced by COVID-19^41^. Fc epsilon RI signaling pathway: Fc epsilon RI interconnecting causes mast cell degranulation and synthesis of proinflammatory mediators^42^. TNF (Tumor Necrosis Factor) signaling pathway: TNF deficit is associated with dysfunctional secretion of inflammatory cytokine, leading to lung pathology and death during respiratory poxvirus infection, and thus TNF is a very significant element for regulating inflammation^43^. Neurotrophin signaling pathway: COVID-19 causes severe brain damage and destruction of the central nervous system derived from neurotrophin^44,45^. Insulin signaling pathway: Obesity-oriented insulin resistance is associated with the induction of proiifnnflammatory macrophage, leads to inflammation of adipose tissue^46^. GnRH (Gonadotropin-Releasing Hormone) signaling pathway: BBB (Blood Brain Barrier) disrupted by a viral infection, lymphocytes (B and T cells), monocytes, and granulocytes can penetrate in the brain parenchyma, which induces inflammation, resulting in dysregulation of GnRH neurons. Additionally, the inflammation of GnRH neurons inhibits GnRH transport through proinflammatory cytokines by impairing the cytoskeleton^47^. Prolactin signaling pathway: HIV patients have greater prolactin quantity compared to others. Besides, prolactin is regarded as a cytokine to stimulate the immune system^48,49^. Adipocytokine signaling pathway: Adipocytokines stimulate inflammation and disrupt immune response, which induces proinflammation in RA (Rheumatoid Arthritis) patients, leading to the development of bone damage^50^. Oxytocin signaling pathway: oxytocin interrupts proinflammatory cytokines' production by inactivating the eIF-2α–ATF4 (Eukaryotic Initiation Factor-2 alpha-Activating Transcription Factor 4) pathway^51^. Relaxin signaling pathway: relaxin inhibitors are good therapeutic targets to suppress inflammation caused by airway dysfunction^52^. AGE-RAGE (Advanced Glycation End product-Receptor of Advanced Glycation End product) signaling pathway in diabetic complications: The binding of AGE to its receptor RAGE can trigger cytokine production, thus, can cause tissue damages, while the blockage of AGE-RAGE can effectively ameliorate the inflammation^53^. Epithelial cell signaling in *Helicobacter pylori* infection: *Helicobacter pylori* interrupts T and B cell signaling to work the immune system. It is apparent that COVID-19 patients with *Helicobacter pylori* might be vulnerable to inflammatory responses^54^.

Generally, SARS-CoV-2 invades the lungs and throat, induces excessive inflammation, which causes cytokines' secretion, resulting in severe complications like acute respiratory failure, pneumonia, and acute liver injury^55^. The leading cause is that the downregulation of ACE2 results in an angiotensin-II (Ang II) increase, which might spur the progression of COVID-19 through activated RAS^56^.

It was discovered that ACE2 is the functional receptor for the SARS-CoV-2 to trigger an infection in the lung alveolar epithelial cells. The internalization of the virus leads to downregulating the ACE2 on the host cell surface that could cause the elevation and demotion of Ang II and angiotensin 1–7 (Ang 1–7), respectively. Such an imbalance between these angiotensins may induce deleterious effects in the lung and heart. Thus, the SARS-CoV-2 affects humans through this mechanism^57–60^. Therefore, RAS blockade may restore the RAS balance by reducing the deleterious effects associated with Ang II^61^. Recent evidence showed that RAS inhibitors might be a promising target for relieving acute-severe pneumonia caused by the COVID-19^62^.

Interestingly, our study identified that the three target proteins (MAPK8, MAPK10, and BAD) are mainly associated with the RAS signaling pathway. MAPK8 and MAPK10 are members of the MAPK family which are the key mediators of inflammation, vasoconstriction, and thrombosis. Besides, overwhelming heart and lung injury in COVID-19 infection might be due to the overactivation of MAPK^63^. Therefore, these proteins' inactivation can also be a viable strategy for relieving COVID-19 induced organ injury. In addition, disposal of inflammatory cells by promoting cell death can be an innovative approach to control excessive inflammation. In this regard, the anti-apoptotic Bcl-2 gene's inhibition can also be a potential target to lessen inflammation^64,65^. Our findings also explored that MAPK8 MAPK10 and BAD proteins are related to three, 12, and two NSAIDs, respectively. During the molecular docking analysis, 6MNA, Rofecoxib, and Indomethacin revealed promising binding affinity along with the highest docking score against MAPK8, MAPK10 and BAD proteins, respectively. The result suggested that the three NSAIDs' key mechanism against COVID-19 might be to inhibit inflammation of lung cells by inactivating the RAS signaling pathway, and blockers of MAPK8, MAPK10 and BAD might suppress cytokine storm. Among various NSAIDs, Indomethacin is a current drug of interest to clinicians. Primary care physicians (New York) reported that Indomethacin had been prescribed to a large number of COVID-19 patients and observed quick recovery from cough, pain, and other symptoms. Such improvements and well-being benefits were not evident in the case of ibuprofen and hydroxychloroquine implementation ( Little^66^). Notably, many researchers previously reported varying degrees of Indomethacin antiviral activity against herpesvirus^67^, pseudorabies virus^68^, cytomegalovirus^69^, hepatitis B virus^70^, vesicular stomatitis virus^71^, rotavirus ^72^, and canine coronavirus^18^. In contrast, 6MNA (an active metabolite of Nabumetone) and Rofecoxib are also potential anti-inflammatory drugs, but studies disclosed that they are less potent than Indomethacin^73,74^. A clinical study recently demonstrated that Indomethacin has potent anti-inflammatory (decrease in IL-6) and antiviral efficacy; taking SRF (Sustained Release Formulation) with 75 mg twice a day achieved full effects in 3 days for patients infected by COVID-19^75^.

Hence, such compelling outcomes indicate that Indomethacin can be considered alone or in combination for antiviral therapy, which may assist in combating human coronavirus (SARS-CoV-2). However, there are also some limitations to our analysis. This study has provided a predictive viewpoint of NSAIDs' mechanism against COVID-19 through public databases. Thus, further experimental results should be validated to achieve the reliability of predicted outputs through in vitro and in vivo followed by NGS (Next Generation Sequencing) technique. Finally, our analysis did not consider the target gene expression level practically after treating the selected NSAIDs (6MNA, Rofecoxib, and Indomethacin), which should be considered and implemented in the future. The proposed (three) NSAIDs against COVID-19 might be significant for clinical application, mainly depending on the genetic, ethnic, and underlying diseases associated with the therapeutic method.

## Conclusion

This study suggests that 6MNA, Rofecoxib, and Indomethacin are the most potent NSAIDs against COVID-19. The basis of this research is an understanding of how these NSAIDs (which stimulates anti-inflammatory processes against COVID-19) work against COVID-19 patients. That scientific evidence informs the selection of NSAIDs, in turn, provides for clinical design against COVID-19. Our research suggests that BAD-Indomethacin's inhibition with the other two hub proteins, MAPK8-6MNA, MAPK10-Rofecoxib might play cumulative actions by inactivating the RAS signaling pathway against COVID-19. Most recently, the efficacy of Indomethacin against COVID-19 has been approved clinically. Our study presents that Indomethacin is a potent therapeutic candidate to relieve COVID-19 symptoms, which is in line with the many previous studies. However, further clinical trial on Indomethacin should be warranted in COVID-19 patients to slow down the progression of SARS-CoV-2 and mitigate the severity.

## Materials and methods

### NSAIDs linked to selected proteins or COVID-19 related proteins

FDA (U.S. Food & Drug Administration) approved NSAIDs (nineteen active drugs and one prodrug) were used in this study. Based on SMILES, targeted gene(s) of NSAIDs were identified through Similarity Ensemble Approach (SEA) (http://sea.bkslab.org/)^76^ and Swiss Target Prediction (STP) (http://www.swisstargetprediction.ch/)^77^ with the "*Homo sapience*" mode^78^. Additionally, COVID-19 targeted proteins were identified by retrieving COVID-19 in PubChem (https://pubchem.ncbi.nlm.nih.gov/). Also, the topological polar surface area (TPSA) value identified by SwissADME is included to verify the NSAIDS' cell permeability; particularly, its permeability is typically limited when the TPSA value is more than 140 Å^2^^79^. The final overlapping proteins between NSAIDs and COVID-19—targeted proteins were identified and visualized by Venny 2.1 (https://bioinfogp.cnb.csic.es/tools/venny/).

### PPI network between NSAIDs and COVID-19 targeted proteins

The protein–protein interaction network (PPI) between NSAIDs and COVID-19 targeted proteins were selected by STRING (https://string-db.org/)^80^ and finally plotted by RStudio.

### Signaling pathway enrichment analysis of overlapping proteins via KEGG database

KEGG database provides correlation of target proteins and signaling pathways through functional annotation^81^. A bubble chart of signaling pathways associated with COVID-19 infection plotted by RStudio. The bubble chart demonstrates a hub signaling pathway (Lowest rich factor) between NSAIDs and COVID-19 related proteins.

### The construction of STN network

The Signaling pathway(s)—Target protein(s)—NSAIDs were used to construct a signaling pathway—target protein—NSAID (STN) network. In this STN network, different colors and shapes (nodes) stand for the signaling pathways (green circle), target proteins (yellow square), and NSAIDs (orange triangles). Gray lines (edges) indicated the interaction of signaling pathways—target proteins—NSAIDs.

The STN networks were utilized to construct a size map, based on degree of values. In this network, green circles (nodes) represented signaling pathways; yellow squares (nodes) represented target proteins, and orange triangles (nodes) represented NSAIDs; its size represented degree value. The size of yellow squares stands for the number of connectivity with signaling pathways; the size of orange triangles stands for the number of connectivity with target proteins. The merged networks were constructed by using RStudio.

### Preparation for MDT of NSAIDs

The ligand molecules were converted .sdf from PubChem into .pdb format using Pymol, and the ligand molecules were converted into .pdbqt format through Autodock.

### Preparation for MDT of target proteins

Three target proteins of a hub signaling pathway, i.e. MAPK8 (PDB ID: 4YR8), MAPK10 (PDB ID: 3TTJ), and BAD (PDB ID: 1G5J), were identified by RCSB PDB (https://www.rcsb.org/). The proteins selected as .pdb format converted into .pdbqt format via Autodock (http://autodock.scripps.edu/).

### NSAIDs- target protein(s) docking

The ligand molecules were docked with target proteins utilizing autodock4 by setting-up 4 energy range and 8 exhaustiveness as default to obtain 10 different poses of ligand molecules^82^. The active site's grid box size was *x* = 20.973 Å, *y* = 25.96 Å and *z* = 41.239 Å. The 2D binding simulation was identified via LigPlot + v.2.2 (https://www.ebi.ac.uk/thornton-srv/software/LigPlus/). After docking, NSAIDs of the lowest binding energy (highest affinity) were selected to visualize the 3D docking simulation in Pymol.

## Supplementary Information

Supplementary Information 1.

Supplementary Information 2.

Supplementary Information 3.

Supplementary Information 4.

Supplementary Information 5.

## Data Availability

All data generated or analyzed during this study are included in this published article (and its “Supplementary Information S1” files).

## Abbreviations

ACE: Angiotensin-Converting Enzyme
ACE2: Angiotensin-Converting Enzyme 2
AGE-RAGE: Advanced Glycation End product /Receptor of Advanced Glycation End product
Angiotensin 1–7: Ang 1–7
Angiotensin-II: Ang II
BAD: Bcl-2-Associated Death promoter
BBB: Blood Brain Barrier
cAMP: Cyclic Adenosine MonoPhosphate
CCL5: C–C Motif Chemokine Ligand 5
CD8^+^ T cells: Cluster of Differentiation 8T cells
cGMP-PKG: Cyclic Guanosine MonoPhosphate-Protein Kinase G
COX-1: Cyclooxygenase-1
COX-2: Cyclooxygenase-2
COVID-19: Coronavirus disease 2019
eIF-2α-ATF4: Eukaryotic Initiation Factor-2 alpha-Activating Transcription Factor 4
ErbB: Erythroblastic Leukemia Viral Oncogene Homolog
FDA: US Food & Drug Administration
FOXO: FOrkhead boX protein O
GnRH: Gonadotropin-Releasing Hormone
HIV: Human Immunodeficiency Virus
IFN-γ: Interferon gamma
IL-4: Interleukin 4
IL-10: Interleukin 10
IL-17: Interleukin 17
KEGG: Kyoto Encyclopedia of Genes and Genomes
MAPK: Mitogen-Activated Protein Kinase
MAPK8: Mitogen-Activated Protein Kinase 8
MAPK10: Mitogen-Activated Protein Kinase 10
MDT: Molecular Docking Test
NF-κB: Nuclear Factor Kappa-light-chain-enhancer of activated B cells
NLR: Nod-Like Receptor
NLRs: Nod-Like Receptors
NSAIDs: Non-Steroidal Anti-Inflammatory Drugs
PPAR: Peroxisome Proliferator-Activated Receptors
PPARα: Peroxisome Proliferator-Activated Receptor-alpha
PPARγ: Peroxisome Proliferator-Activated Receptor-gamma
PPARβ/δ: Peroxisome Proliferator-Activated Receptor-beta/delta
PAMPs: Pathogen-Associated Molecular Patterns
PPI: Protein–protein interaction
RA: Rheumatoid Arthritis
RAS: Renin Angiotensin System
RLR: RIG-I-Like Receptor
RLRs: RIG-I-Like Receptors
SARS-CoV-2: Severe Acute Respiratory Syndrome CoronaVirus 2
SEA: Similarity Ensemble Approach
STN: Signaling pathway-Target protein-NSAID
STP: Swiss Target Prediction
TLR: Toll-like receptor
TLRs: Toll-like receptors
TNF: Tumor Necrosis Factor
TNF-α: Tumor Necrosis Factor-Alpha
TPSA: Topological Polar Surface Area
VEGF: Vascular Endothelial Growth Factor
VEGFA: Vascular Endothelial Growth Factor A
WHO: World Health Organization
WNT: Wingless/Integrated
