# Supplementary material for: Network pharmacology approach to decipher signaling pathways associated with target proteins of NSAIDs against COVID-19
Source: Sci Rep. 2021 May 5;11:9606. doi: 10.1038/s41598-021-88313-5 (PMC8100301; doi:10.1038/s41598-021-88313-5)
Supplement: Supplementary file 1 — Supplementary Information 1. [file 41598_2021_88313_MOESM1_ESM.pdf]

Title Page

Research article

Network pharmacology approach to decipher signaling pathways associated with target proteins of NSAIDs against COVID-19

Ki Kwang Oh[1], Md. Adnan[1], Dong Ha Cho[1*]

[1]Department of Bio-Health Convergence, College of Biomedical Science, Kangwon National University, Chuncheon, 24341, Korea

***Correspondence:** chodh@kangwon.ac.kr; Tel.: +82-33-250-6475

Email of Authors:
[1]Ki Kwang Oh (KKO): nivirna07@kangwon.ac.kr
[1]Md. Adnan (MA): mdadnan1991.pharma@gmail.com
[1]Dong Ha Cho (DHC): chodh@kangwon.ac.kr

Running title: Potential Non-Steroidal Ani-Inflammatory Drugs (NSAIDs) and novel mechanism against SARS-CoV-2

Supplementary Figure S1.

(a) Interactions between three bioactive compounds and MAPK8.

(b) Interactions between twelve bioactive compounds and MAPK10.

(c) Interactions between two bioactive compounds and BAD.

(a)

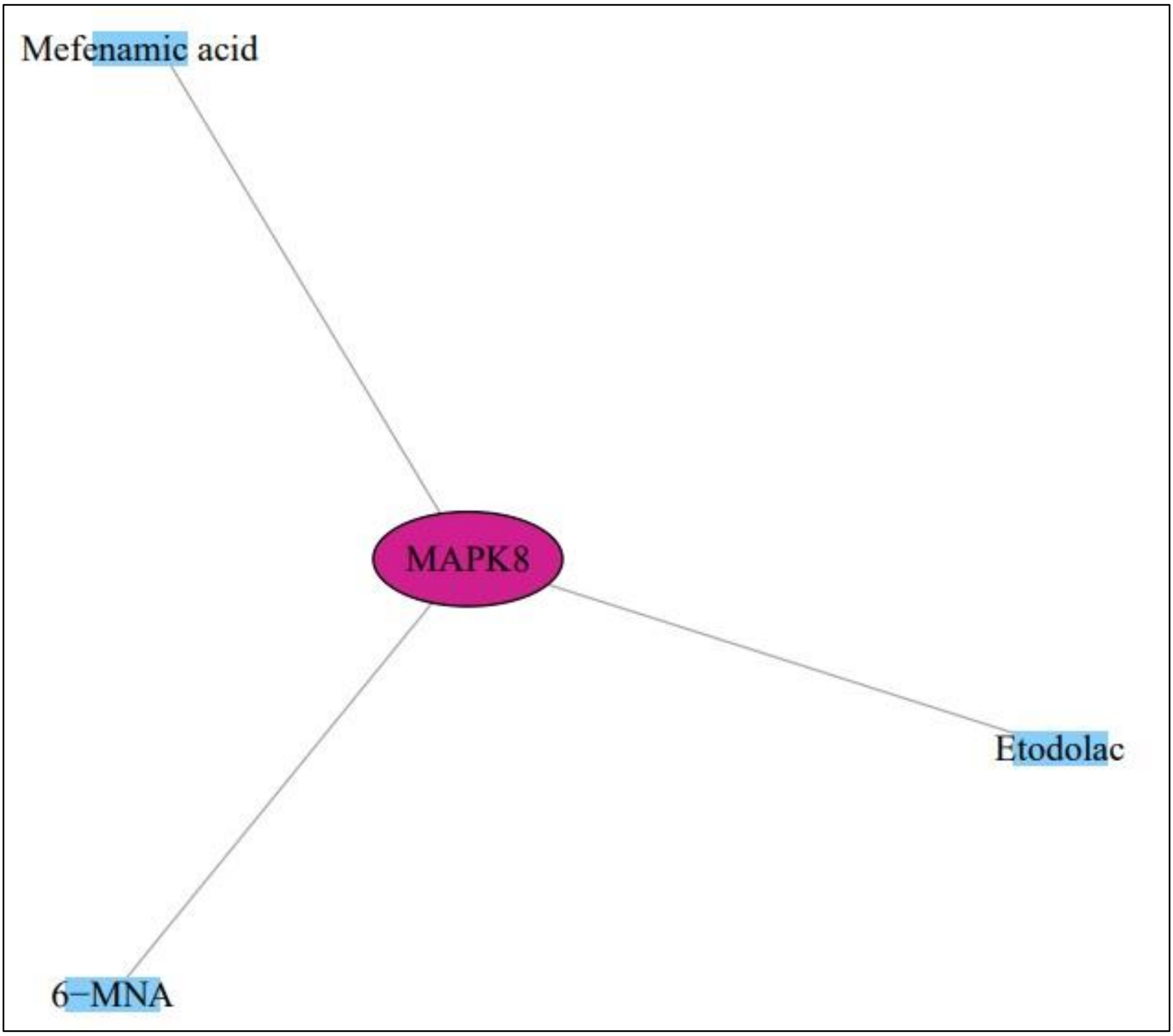

(b)

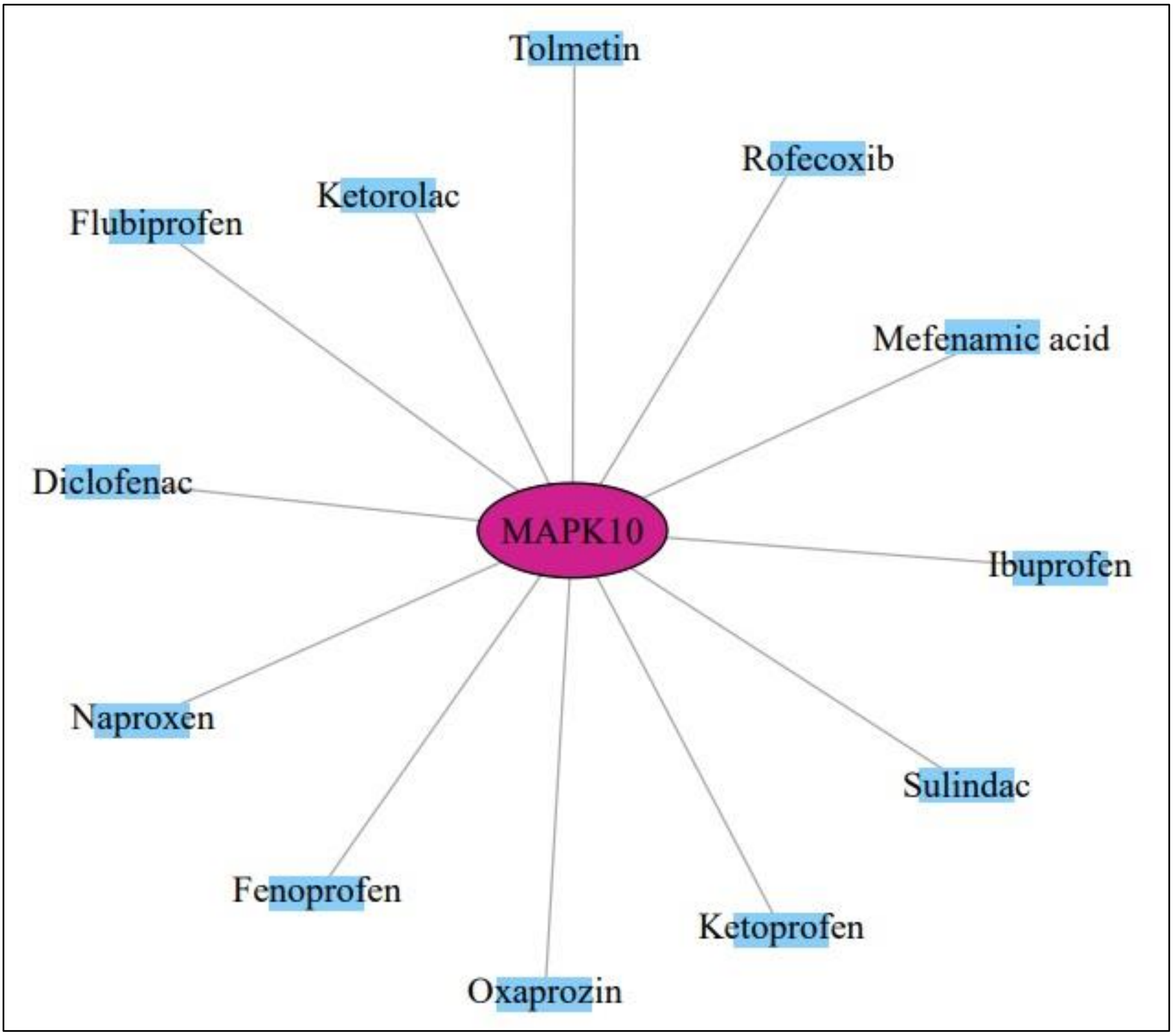
Tolmetin
Rofecoxib
Ketorolac
Flubiprofen
Mefenamic acid
Diclofenac
MAPK10
Ibuprofen
Naproxen
Sulindac
Fenoprofen
Ketoprofen
Oxaprozin

(c)

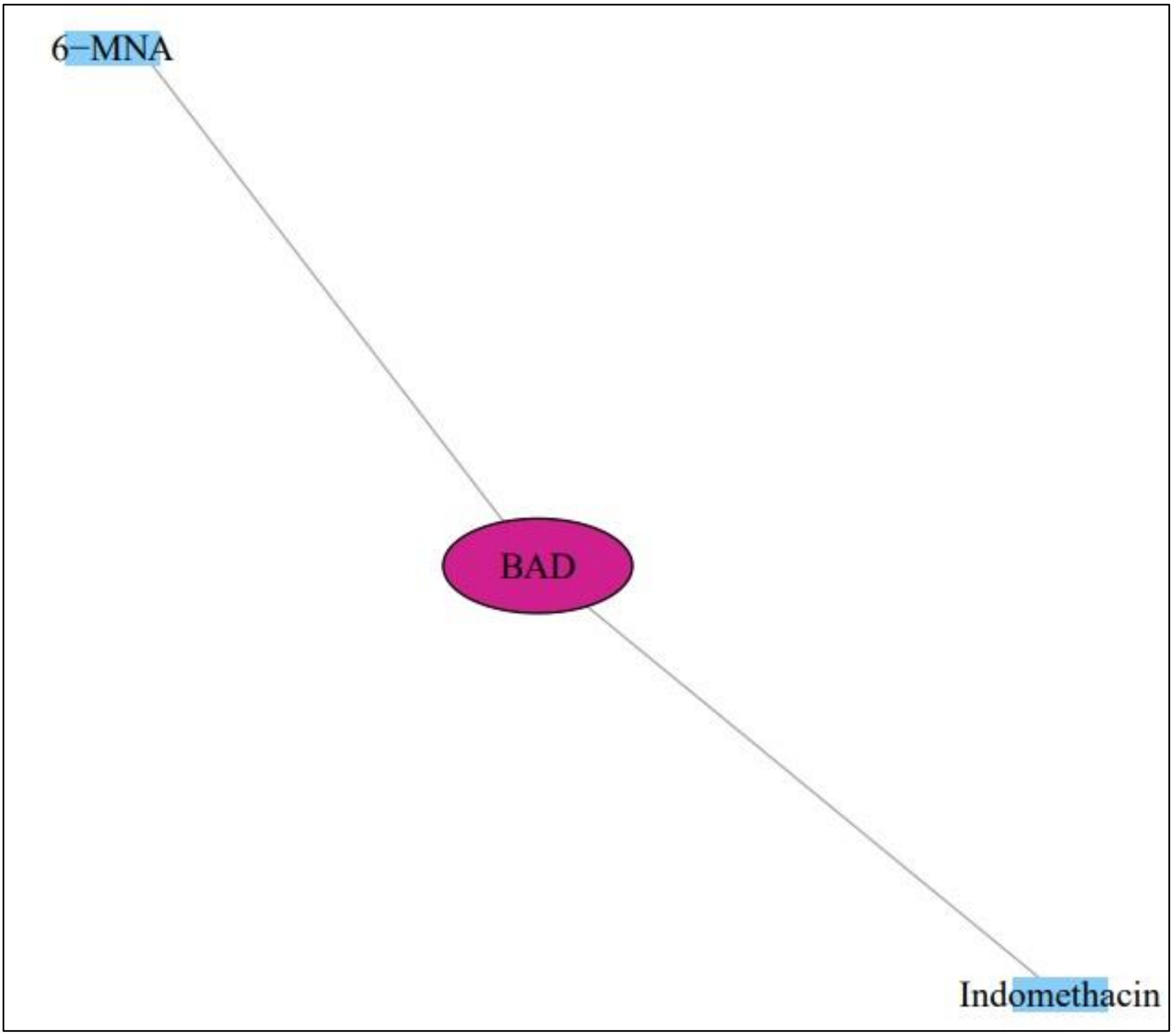
6−MNA
BAD
Indomethacin
