# Supplementary material for: Network pharmacology approach to decipher signaling pathways associated with target proteins of NSAIDs against COVID-19
Source: Sci Rep. 2021 May 5;11:9606. doi: 10.1038/s41598-021-88313-5 (PMC8100301; doi:10.1038/s41598-021-88313-5)
Supplement: Supplementary file 2 — Supplementary Information 2. [file 41598_2021_88313_MOESM2_ESM.pdf]

A total of 781 target proteins related to NSAIDs in both SEA and STP databases
PTGS2
CA9
CA12
CA2
CA5A
CA5B
CA14
CA6
CA4
CA13
CA3
CA7
ADRA2B
PTGES
MAPK14
CYP2D6
SLC6A2
SLC6A3
SLC6A4
PTGS1
CA1
RPA1
CRACR2A
ORAI1
MT-CO2
STIM1
CARM1
RXRA

RARG
F10
NR3C2
CCKAR
EPHX2
NPY5R
CHRNA7
FPR1
SLCO1B1
SLC5A1
PPP1CA
RARB
CXCL8
TTR
AKR1C3
SLC22A6
AKR1B10
CXCR1
CYP2C9
AKR1C2
SFN
KCNQ2
FTO
FABP2
CXCR2
PTGER3
KCNQ4
PYGL
CTSA

GRIK1
SLC15A1
TXNRD1
AKR1C1
KCNJ5
TAS1R2
SELP
RPS6KB2
MMP16
KCNJ3
EPHB3
TAS1R3
FABP5
FABP4
C1R
SLC22A12
S100B
SELL
KCNQ1
THRA
GPR139
CDC42
AKR1B1
P2RX7
PTGDR2
FEN1
THRB
CASP6
EGLN3

TAAR1
KCNJ6
FABP3
LPAR2
PLA2G1B
PTPRG
KCNK9
TRPV1
ERCC5
IDO1
DHODH
EIF4E
HSD3B2
PRKAB2
POLI
ALOX5AP
HSD17B1
KMO
CES2
MPG
POLH
NR0B2
PTPN22
HSD17B14
HSD17B2
FUT7
GSTA1
NOTUM
PRKAB1

MPL
PRKAG1
PHLPP2
KCNMA1
ACVR2B
PTGER1
ESR2
EGLN2
SLC2A4
FFAR1
GLI1
ACPP
LTA4H
FDFT1
ACACA
MTNR1B
CPA1
ACE2
SLC7A5
GPR88
PPARA
PPARG
ALOX5
EGLN1
MTNR1A
CPN1
PRKG2
PRKG1
MME

FAAH
GRK3
LPAR5
PTGER4
KYNU
SCN4A
CAPN2
AOC3
NR1H3
MGLL
PPARD
ADRB3
FFAR4
MMP2
CCR8
CACNA1A
CACNB1
MMP9
ACACB
METAP1
FNTB
GCK
MMP13
PRKX
SGK2
FNTA
F13A1
LPAR3
GAPDH

PKN2
PLA2G10
MCL1
KAT2A
PRKACA
SRD5A2
CETP
NR1H2
TSPO
RHOA
ACE
CHRNG
CES1
ROCK1
GABRB1
LPAR1
CHRNB1
CDC42BPA
CHRND
CASR
RPS6KA5
DAGLA
CFTR
MMP14
CASP1
EPHX1
ADAMTS4
CHRNA1
S1PR2

NEK4
ST6GAL1
TAS1R1
GSK3A
MMP12
MMP3
BAD
PRKCG
AKT3
MMP8
CYP2C19
LCT
KCNN4
SI
GBA2
MMEL1
UGCG
ADAM12
GRIA4
PRCP
PTPN13
MGAM
CT니
PTPRC
CD274
CTSK
CTSB
GBA
SMAD3

CTSS
NTSR1
HMGCR
PDK2
PTGES2
SLC1A1
CPA3
PDYN
DNPEP
RNPEP
YARS
ANPEP
NAALAD2
LAP3
TH
CSK
FOLH1
TPH1
FCER2
PAM
CPB2
CPB1
S1PR5
NPPA
SOAT2
MMP1
SLC7A11
S1PR4
ENPEP

SLC22A8
TBXAS1
AR
PLA2G2A
PLA2G4A
DUSP2
PTGIR
EWS-Fli1
KLK14
AKR1C4
CPT1B
CPT1A
PTGDR
CYSLTR2
KLK5
ACR
SENP1
LTC4S
NQO2
GPR183
HTR6
NPBWR1
BAZ2A
BAZ2B
SSTR5
SPR
CYSLTR1
GRIK2
TUBB1

OXER1
ELAVL1
TBXA2R
ACER2
SLC18A2
CHRNB4
SLC9A2
CHRNA3
PLAUR
TRPM8
KCNK3
STS
CHRNA4
CHRNB2
KDM2A
ELANE
BCL2A1
ADIPOR1
ADIPOR2
L3MBTL3
PHGDH
SLC6A1
SLC6A7
MPO
CYP1A2
CXCR5
HCAR2
SORT1
DDX3X

RXFP1
NR4A1
MAPK8
TRPM2
RCE1
KDM4C
CTBP2
LIG1
RBP4
KDM6B
HIPK2
AGER
GFER
RAPGEF4
NR1H4
CTSV
NPSR1
DAPK3
PMM2
KDM4D
CSNK1A1
STK17A
KDM4E
IDE
MAPK10
SENP7
UCHL1
DYRK4
FES

MATK
ATG4B
CDC7
PAK4
MAP4K2
CSNK2A2
GRM4
CDK5
CYP26A1
HSD17B10
CYP1B1
ABCG2
NFE2L2
RYR2
UTS2R
SLC16A3
ITGA3
TOP2B
CYP11B1
RELA
TAS2R14
PDK4
CYP1A1
CYP11B2
KDM2B
MMP7
PDK3
BMP1
EDNRA

EDNRB
GRK6
MAOB
NFKB1
RBBP9
NCEH1
APP
MAPT
TNFRSF1A
KLF5
HNF4A
CALM1
CYP17A1
PHF8
MITF
SELE
STAT1
PLCG2
PRNP
G6PD
ALPL
PLAU
NQO1
LTB4R2
CACNA1G
CDC25C
LTB4R
APEX1
GPR17

MMP10
PRKAA2
CDC25A
PIN1
SIRT5
DBF4
CDC25B
SLC40A1
GRM5
SMN1
SMN2
CTSF
UQCRB
ATR
ATM
GLRA1
BDKRB2
NLRP1
GABRA5
BRD2
NR4A2
BRD3
BRD4
TAF1L
MLYCD
MCOLN3
DKK1
LDHB
CACNA1C

LDHA
TTK
CYP27A1
AGBL2
HPSE
SLC5A4
PRUNE1
QTRT1
PLA2G4B
CACNA1B
SLC6A11
G6PC
PTPN1
SLC1A5
KEAP1
UBE2I
HDAC1
CYP26B1
ACHE
HDAC8
CYP3A4
SGMS1
PDE4D
CYP24A1
SOAT1
GPBAR1
HDAC6
PDE4B
CSNK1D

AHR
WNK1
PTPN2
SIGMAR1
KCNA5
SLC2A1
CTDSP1
MAOA
APOBEC3A
ADAM17
PTPN6
SENP6
GLS
ABCB1
SLC5A2
ERAP2
CAPN1
SLC13A5
IGFBP5
PTGER2
RNASEH1
GRIN2B
HDAC2
CBFB
NLRP3
ASAH1
P4HTM
CHRM5
KARS

CASP7
GLO1
DGAT1
PLAT
FLT3
ADORA3
EP300
AADAT
CLK1
SCN3A
CCR3
PTPRB
MAPK9
FOS
APOBEC3G
HDAC3
MIF
BCHE
HSD17B3
JUN
CASP3
CNR1
SENP8
FPR2
CNR2
GPR55
HKDC1
PAX8
PDGFRB

CYP19A1
MCHR1
DYRK2
GRM2
CHRM1
PRSS1
GSK3B
TNIK
PDPK1
GABRB3
TYMS
LRRK2
MTOR
CCND1
CCNE2
CDK4
KIF11
SHH
FLT1
HRH1
HRH4
PIK3CA
S1PR1
PDE5A
WNT3A
AXIN2
PARP1
PARP2
TNKS2

TNKS
AURKB
AGPAT2
GABRA2
PIM1
GABRB2
GABRG2
GABRA3
GABRA1
CDK2
CCNE1
FABP1
MAPK1
HSPA1A
BCAT2
DYRK1A
CSNK2A1
GRK2
ECE1
ABCC1
PLEC
CCNC
KDM4A
PDE7A
MKNK2
MAP2K1
KDM5C
DAO
AKR1A1

MAP3K8
PARP15
PARP10
PYGM
ITGB1
METAP2
PDE9A
ITGAV
MKNK1
ICAM1
GSTP1
HAO2
CDK8
ITGA5
ITGA2
ERN1
PKM
ESR1
CREBBP
NEK2
ITGAL
GSR
PPP5C
DHFR
MDM2
OPRK1
PSEN2
OPRL1
OPRM1

IMPDH2
ADAMTS5
EGFR
PIK3CD
PIK3CB
TDO2
PTPRF
GPR35
CMA1
CTSG
SCN10A
VCP
ADORA1
DUSP3
PSENEN
NCSTN
APH1A
PSEN1
APH1B
ITGA4
ITGB3
ITGB2
ITGA1
PGR
EPRS
HSP90AA1
CISD1
SRD5A1
KDM5A

KDM5B
PDE10A
SLC16A1
ABAT
YWHAG
CCR2
PIM2
PDE4A
CACNA2D1
CAMKK1
CAMKK2
PIM3
HAO1
TOP1
NOS2
BCL2L1
MC1R
CD38
MC4R
PLA2G2E
PLA2G2D
PLA2G5
HSD11B1
TNNC1
TNNT2
TNNI3
BCL2
GRIN1
CTSL

AGTR2
AMPD3
PFKFB3
FGFR1
KDR
TYK2
TERT
CDK5R1
PIK3CG
ADORA2B
PTK2B
VEGFA
SRC
ALOX12
ABL1
PRKDC
HCK
PI4KB
EPHB4
RAF1
DPP4
CHEK1
ADORA2A
HTR2C
CSF1R
MAPKAPK2
RPS6KA3
MALT1
RPS6KB1

AURKA
SIRT1
PSMB5
IKBKB
JAK3
JAK1
JAK2
ROCK2
IGF1R
MAPK3
VCAM1
SYK
PRKCA
TDP2
PREP
HASPIN
CDK9
CHEK2
NISCH
CLK4
CLK2
QPCT
DYRK3
PPIA
CSNK1G1
RPS6KA1
NOS1
ITK
PABPC1

CCR5
TYMP
KIT
FLT4
CCNT1
ENPP2
GYS1
MET
CTNNB1
CNOT7
ALK
IDH1
DRD1
PGGT1B
ROS1
DYRK1B
LIPE
TKT
F2
HMOX1
STAT3
CYP2A6
CTSC
ALDH3A1
AKT1
HSP90B1
RORC
DRD2
KCNA3

PIP4K2C
PLG
KLKB1
CCNB3
TGFBR1
CDK1
CCNB1
CCNB2
PLK1
ITGB7
MARS
HSD11B2
PDE4C
KDM3A
TEK
HIF1A
HTR2B
HTR7
NOS3
C5AR1
MAP3K7
BRS3
TLR9
S1PR3
AGTR1
DCTPP1
CCNA1
CCNA2
