# Supplementary material for: Network pharmacology approach to decipher signaling pathways associated with target proteins of NSAIDs against COVID-19
Source: Sci Rep. 2021 May 5;11:9606. doi: 10.1038/s41598-021-88313-5 (PMC8100301; doi:10.1038/s41598-021-88313-5)
Supplement: Supplementary file 3 — Supplementary Information 3. [file 41598_2021_88313_MOESM3_ESM.pdf]

228 overlapped target proteins between SEA and STP
PTGS2
CA9
CA12
CA2
CA5A
CA5B
CA14
CA6
CA4
CA13
CA3
CA7
ADRA2B
PTGES
MAPK14
CYP2D6
SLC6A2
SLC6A3
SLC6A4
PTGS1
CA1
RPA1
MT-CO2
RXRA
NR3C2
CCKAR
EPHX2
NPY5R

SLCO1B1
PPP1CA
CXCL8
TTR
AKR1C3
SLC22A6
AKR1B10
CXCR1
AKR1C2
FABP2
PTGER3
PYGL
CTSA
GRIK1
AKR1C1
FABP5
FABP4
C1R
SLC22A12
THRA
GPR139
CDC42
AKR1B1
P2RX7
PTGDR2
FEN1
THRB
EGLN3
FABP3

PLA2G1B
PTPRG
ERCC5
IDO1
DHODH
ALOX5AP
KMO
CES2
GSTA1
NOTUM
KCNMA1
PTGER1
ESR2
FFAR1
FDFT1
MTNR1B
CPA1
ACE2
PPARA
PPARG
ALOX5
EGLN1
MTNR1A
MME
FAAH
PTGER4
AOC3
NR1H3
PPARD

FFAR4
MMP2
MMP9
FNTB
GCK
MMP13
FNTA
LPAR3
PKN2
PLA2G10
MCL1
SRD5A2
CETP
NR1H2
RHOA
ACE
CES1
ROCK1
LPAR1
MMP14
CASP1
ADAMTS4
GSK3A
MMP12
MMP3
BAD
PRKCG
MMP8
KCNN4

ADAM12
CTSK
CTSB
CTSS
NTSR1
ANPEP
FOLH1
PAM
CPB2
CPB1
MMP1
ENPEP
TBXAS1
AR
PLA2G2A
PLA2G4A
PTGIR
AKR1C4
PTGDR
NQO2
TUBB1
OXER1
TBXA2R
TRPM8
STS
KDM2A
ELANE
MPO
CYP1A2

HCAR2
SORT1
NR4A1
MAPK8
KDM4C
CTBP2
LIG1
RBP4
KDM6B
RAPGEF4
CTSV
KDM4D
CSNK1A1
KDM4E
IDE
MAPK10
DYRK4
CDC7
CSNK2A2
GRM4
CDK5
CYP26A1
ABCG2
SLC16A3
CYP11B1
CYP11B2
KDM2B
MMP7
BMP1

EDNRA
EDNRB
MAOB
HNF4A
CYP17A1
SELE
PLAU
APEX1
CDC25A
PIN1
DBF4
CDC25B
GRM5
GLRA1
GABRA5
BRD2
BRD3
BRD4
MLYCD
LDHA
PTPN1
CYP26B1
ACHE
PDE4D
PDE4B
CSNK1D
PTPN2
KCNA5
SLC2A1

MAOA
ADAM17
ABCB1
CAPN1
PTGER2
HDAC2
ASAH1
GLO1
DGAT1
FLT3
ADORA3
EP300
CLK1
HDAC3
MIF
BCHE
CASP3
CNR1
CNR2
PDGFRB
CYP19A1
MCHR1
DYRK2
CHRM1
PRSS1
GSK3B
