# Supplementary material for: Network pharmacology approach to decipher signaling pathways associated with target proteins of NSAIDs against COVID-19
Source: Sci Rep. 2021 May 5;11:9606. doi: 10.1038/s41598-021-88313-5 (PMC8100301; doi:10.1038/s41598-021-88313-5)
Supplement: Supplementary file 4 — Supplementary Information 4. [file 41598_2021_88313_MOESM4_ESM.pdf]

**COVID 19-related target proteins : 466**
POLR2G
KEAP1
ALB
ATP6AP1
BACH1
BLVRA
BLVRB
CAT
CPOX
EDN1
EDNRA
ELN
FECH
FTH1
FTL
GPX1
GPX4
GSR

GTF2B
GTF2F2
HMOX1
IDE
INS
LOXL1
MAF
MAT1A
MAT2A
NFE2L2
SLC11A2
NUP88
PRDX1
POLR2B
POLR2E
SOD1
SOD2
PRDX2
TF

TFAM
TFRC
UROD
UROS
EIF4H
BAG6
NUP214
NCOA4
AIFM1
COPS2
ABCG2
PRDX6
NUP58
DCAF7
SIGMAR1
FBLN5
TXNRD2
ATP5MG
NEK6

COPS8
PRDX3
COPS6
COPS5
SPART
NUP210
SIRT3
NUP62
PRDX5
TXN2
MAT2B
DLL1
FLVCR1
SLC40A1
COPS7A
SLC15A3
NUP54
CYCS
STEAP3

DNAJC11
CAND1
UBQLN4
MCOLN1
MIB1
ATP13A3
ZNF503
NEK9
NEK7
ALG11
SLC6A19
RPL10A
CCL3
AGT
AGTR1
AGTR2
NR0B1
AKT1
ANPEP

ANXA1
APAF1
FASLG
ATF4
BAD
BAX
BCL2
BCL2L1
BDKRB1
BDKRB2
BID
BSG
BST2
C1QA
C1QB
C1QC
LDLRAD4
CASP1
CASP3

CASP7
CASP8
CASP9
CBFB
CD2
CD3E
CD3G
CD247
CD4
CD8A
CD8B
CEBPA
CEBPB
CEBPD
CEBPE
CEBPG
CFTR
CHUK
CMA1

CCR5
CPA3
CPB1
CPB2
ATF2
CRP
MAPK14
CTSD
CTSG
CTSL
CTSZ
CYP11B2
ACE
DDIT3
DDX1
S1PR1
S1PR3
EIF1AX
EIF2S1

EIF2S3
EIF4E
ENPEP
ERN1
FABP2
FCGR1A
FCGR2A
FCGR2B
FCGR3A
FOS
MTOR
FYN
GP1BA
NR3C1
GSTA2
GSTM1
GZMH
HDAC1
HLA

HMGB1
IFI27
IFNAR1
IFNAR2
IGHE
IGHG1
IGHG2
IGHG4
IGLC2
IGLL1
IKBKB
IL1B
IL2
IL2RA
IL6
IL6R
IL6ST
CXCL8
IL17A

CXCL10
EIF3E
IRF3
ITGAL
JAK1
JUN
JUNB
KNG1
KPNA2
RPSA
LCK
SMAD2
SMAD3
MAS1
MCL1
MAP3K4
NR3C2
MME
MYD88

PPP1R12A
PPP1R12B
NEDD4
NFKB1
NFKBIA
NOS2
NOS3
OAS1
OAS2
OAS3
OPRD1
OPRM1
PA2G4
FURIN
PHB
PIK3C3
PPARA
PPARG
PPIA

PPP1CA
PPP1CB
PPP1CC
PPP1R1A
PPP1R2
PPP1R3A
PPP1R3C
PPP1R3D
PPP1R7
PPP1R8
PPP1R10
MAPK1
MAPK3
MAPK8
MAPK11
MAPK9
MAPK10
MAPK13
MAP2K6

MAP2K7
EIF2AK2
PTGS1
PTGS2
RAF1
RELA
REN
RPL3
RPL4
RPL5
RPL6
RPL7
RPL7A
RPL8
RPL9
RPL11
RPL12
RPL13
RPL15

RPL17
RPL18
RPL18A
RPL19
RPL21
RPL22
RPL23A
RPL24
RPL26
RPL27
RPL30
RPL27A
RPL28
RPL29
RPL31
RPL32
RPL34
RPL35A
RPL37

RPL37A

RPL38

RPL39

RPL41

RPL36A

RPLP0

RPS2

RPS3

RPS3A

RPS4X

RPS5

RPS6

RPS6KA1

RPS6KA2

RPS6KA3

RPS7

RPS8

RPS9

RPS10

RPS11
RPS12
RPS13
RPS14
RPS15
RPS15A
RPS16
RPS17
RPS18
RPS19
RPS20
RPS21
RPS23
RPS24
RPS25
RPS26
RPS27
RPS27A
RPS28

RPS29
S100A7
MAPK12
CCL2
MAP2K4
SGTA
STAT1
STAT2
MAP3K7
ELOC
ELOB
PPP1R11
TGFB1
TGFBR1
TGFBR2
THBD
TMPRSS2
TNF
TRAF3

TRAF6
TYK2
UBA52
VEGFA
XBP1
ZAP70
ULK1
CUL2
IKBKG
EIF3A
EIF3B
EIF3C
EIF3D
EIF3F
EIF3H
EIF3I
BECN1
S1PR4
MBTPS1

RIPK1
FADD
NR1I2
NAE1
EIF2S2
UBA3
RPL14
FCGR2C
ATG12
RPL23
EIF2AK3
ISG15
IKBKE
ULK2
ATG13
RB1CC1
RBX1
BCL2L11
CEBPZ

ATP6AP2
CALCRL
EIF1
IRF9
PRG3
RACK1
EIF3M
ATG7
RPL35
PHB2
ATF6
PPP1R13B
RPL13A
PADI4
DDX58
PPP1R15A
IL17RA
RPL36
PPP1R16B

PPP1R14B
BBC3
EIF3K
TRBC1
TRAC
PYCARD
TBK1
PIK3R4
TLR7
MBTPS2
EIF3L
ZFYVE1
S1PR5
TLR9
PPP1R12C
PPP1R14D
ATG16L1
WIPI1
PPP1R9A

TSR1
NKRF
PMEPA1
MAVS
ACE2
IFIH1
MMP25
MPP5
ATG3
PPP1R3B
ZYG11B
PPP1R14C
ATG10
EIF2A
PPP1R1B
MAP1LC3A
PPP1R9B
IL17RC
AOPEP

PPP1R15B
PPP1R16A
PPP1R3F
PPP1R3E
PPP1R14A
IL17F
NLRP3
CCDC124
ATG4A
RPL10L
TICAM1
PPP1R1C
PIKFYVE
TUBB
TPCN2
TREML4
PTPRS
PPP1R3G
E

MMP5
S
ORF3a
M
ORF6
ORF7a
ORF7b
N
ORF10
ORF8
ORF1ab

target proteins
