# Supplementary material for: Network pharmacology approach to decipher signaling pathways associated with target proteins of NSAIDs against COVID-19
Source: Sci Rep. 2021 May 5;11:9606. doi: 10.1038/s41598-021-88313-5 (PMC8100301; doi:10.1038/s41598-021-88313-5)
Supplement: Supplementary file 5 — Supplementary Information 5. [file 41598_2021_88313_MOESM5_ESM.pdf]

The final 26 overlapping target proteins
PTGS2
MAPK14
PTGS1
NR3C2
PPP1CA
CXCL8
FABP2
ACE2
PPARA
PPARG
MME
MCL1
ACE
CASP1
BAD
ANPEP
CPB2
CPB1
ENPEP
MAPK8
IDE
MAPK10
ABCG2
CYP11B2
EDNRA
CASP3
